# Influence of temperature on daily locomotor activity in the crab *Uca pugilator*

**DOI:** 10.1371/journal.pone.0175403

**Published:** 2017-04-26

**Authors:** Audrey M. Mat, Gideon P. Dunster, Valerio Sbragaglia, Jacopo Aguzzi, Horacio O. de la Iglesia

**Affiliations:** 1 Department of Biology, University of Washington, Seattle, Washington, United States of America; 2 Marine Science Institute, (ICM-CSIC), Barcelona, Spain; McGill University, CANADA

## Abstract

Animals living in the intertidal zone are exposed to prominent temperature changes. To cope with the energetic demands of environmental thermal challenges, ectotherms rely mainly on behavioral responses, which may change depending on the time of the day and seasonally. Here, we analyze how temperature shapes crabs’ behavior at 2 different times of the year and show that a transition from constant cold (13.5°C) to constant warm (17.5°C) water temperature leads to increased locomotor activity levels throughout the day in fiddler crabs (*Uca pugilator*) collected during the summer. In contrast, the same transition in environmental temperature leads to a decrease in the amplitude of the daily locomotor activity rhythm in crabs collected during the winter. In other words, colder temperatures during the cold season favor a more prominent diurnal behavior. We interpret this winter-summer difference in the response of daily locomotor activity to temperature changes within the framework of the circadian thermoenergetics hypothesis, which predicts that a less favorable energetic balance would promote a more diurnal activity pattern. During the winter, when the energetic balance is likely less favorable, crabs would save energy by being more active during the expected high-temperature phase of the day—light phase—and less during the expected low-temperature phase of the day—dark phase. Our results suggest that endogenous rhythms in intertidal ectotherms generate adaptive behavioral programs to cope with thermoregulatory demands of the intertidal habitat.

## Introduction

Marine organisms living in the intertidal zone occupy a complex temporal environment. Their exposure to the solar and lunar cycles is associated with prominent environmental oscillations, including the light-dark (LD) and tidal cycles [1, 2]. As in many intertidal species, several rhythms have been studied in the crab genus *Uca*, including circatidal, circadian, and circalunar rhythms [3, 4]. *U*. *pugilator* (Bosc, 1802) is a sand fiddler crab living on the east coast of the USA, from Massachusetts to Florida, in the Gulf of Mexico up to Mississipi, and in the Bahamas [5, 6]. Immediately after collection from the beach, *U*. *pugilator* displays both a circatidal and a circadian rhythm of locomotor activity under LD cycles or constant illumination [7, 8].

Besides the cyclic ebb and flow of water and LD alternations, intertidal ectotherms are also exposed to prominent temperature variations. In the northern areas of its geographic range, *U*. *pugilator* can face subfreezing temperatures [6]. On the other extreme of the thermal spectrum and depending on the latitude, they can be exposed to up to 40°C [9, 10]. Within the same beach, intertidal crabs are exposed to high amplitude tidal and daily oscillations in ambient temperature; and indeed temperature cycles can act as a Zeitgeber, an environmental cycle that ‘entrains’ biological clocks [3]. Entrainment of both physiological and behavioral rhythms by temperature may in turn favor thermoregulatory processes in anticipation to temperature challenges. For instance, the chromatophore dispersion rhythm in fiddler crabs impacts the absorption of solar radiation, which is increased by darker colorations [9, 11]. On the other hand, behaviors like retreating to a burrow can constitute a thermal shelter to limit direct exposure to extreme temperatures [12]. The ‘circadian thermoenergetics hypothesis’ proposes that thermoenergetic demands likely have a strong selective pressure for specific daily and circadian behavioral programs [13–15]. According to this hypothesis, metabolic challenges such as decreased environmental temperature and energy sources, or conversely, increased locomotor activity demands to obtain food, tend to favor activity during the warmer times of the day and rest during the colder times of the day. Although this hypothesis is supported by several studies in homeotherms, the possibility exists that similar trade-offs exist for the daily activity programs of ectotherms.

Rhythms of spontaneous locomotor activity in the laboratory are used as a proxy for rhythmic behaviors in the wild, and are expected to show responses to environmental temperatures that may have adaptive significance. In the present study, we analyze the effect of constant environmental temperature on the daily locomotor activity rhythm of *U*. *pugilator* males and show that the response of their locomotor activity rhythm to a temperature shift differs between winter and summer. As predicted by the circadian thermoenergetics hypothesis, crabs during the winter show increased diurnality (activity during the light phase of the LD cycle) in response to a constant lower environmental temperature. Our results suggest that the selective pressure for specific circadian and daily behavioral programs by thermoenergetic demands may have acted in homeotherms as well as ectotherms.

## Materials and methods

The experiments were performed at the University of Washington, Seattle (WA, USA) on *U*. *pugilator* males. Three batches of animals, one for each experiment, were purchased from Carolina Biological Supply Company (NC, USA). More crabs were purchased in August to test them under free-running conditions. All 3 batches were collected from the same geographical region: salt marshes in northern Florida. Crabs were maintained in the vendor’s laboratory for 1–3 weeks at 18°C and then shipped to our laboratory where experiments started after 2–3 days of acclimation. For the experiments, crabs were kept in a temperature- and humidity-controlled chamber, placed in individual cells (12cm diameter) in the same aquarium. Cells contained approx. 3 mm of seawater (pH = ~7.00 at 13°C). The aquarium was continuously maintained under a flow of seawater (2 Lxmin^-1^) from a 120L reservoir. Every 3–4 days, 30L of water from the reservoir were replaced with fresh seawater. Crabs were not fed during the experiments to avoid disturbing activity patterns.

### Temperature experiments

Temperature experiments were performed in March 2014 (n = 27 crabs), August-September 2014 (referred to as “August” from now on, n = 22) and March 2015 (n = 28). The temperature of the chamber was initially set to 13.5 ± 1°C (cold) for 6–10 days, then increased over 10 h to reach 17.5 ± 1°C (warm) and maintained at this temperature 6–10 days. For the August 2014 and March 2015 experiments the temperature was then brought back to 13.5 ± 1°C for 9 days (this last phase of the experiment could not be completed for March 2014 due to technical problems with the chamber). Animals were exposed to a 12:12 LD cycle (~100:1 lux) throughout the experiments unless otherwise specified. Water temperature was recorded every 15 minutes (ibutton DS1922L, Maxim Integrated, California, USA).

### Free-running experiments

Free-running experiments were done from September to November 2014. In September-October 2014, 17 crabs were kept at 17 ± 1°C under a 12:12 LD cycle for 10 days then released into constant light (LL) for 12 days. In November 2014, 11 crabs were kept at 13 ± 1°C under a 12:12 LD cycle for 8 days and then released into LL for 12 days.

### Light exposure

Crabs were continuously exposed to LED-generated dim red light plus infrared light (the latter to allow video-recording during the dark phase; LEDwholesalers, CA, USA). During the photophase, light was additionally provided with LEDs in the visible spectrum (LEDwholesalers, CA, USA). The LEDs were connected to an Arduino board (Arduino Uno, www.arduino.cc), controlled *via* a script ran on the Arduino software (version 1.0.5) and synchronized by a real-time clock for accuracy (ChronoDot V2.1 High Precision RTC, macetech LLC, CA, USA).

### Behavioral data acquisition, processing and analysis

The behavior of the crabs was measured using an infrared-sensitive camera (model UI-1220LE-M-GL, IDS Imaging Development Systems GmbH, Obersulm, Germany). The camera was interfaced with Yawcam (http://www.yawcam.com, version 0.4.1). A picture of the whole aquarium was taken every 10 sec from the top.

Behavioral analysis was based on locomotor activity using a script in Matlab (version 2013a) that recognizes every individual on each picture and calculates the Euclidian distance covered by each crab between two consecutive pictures as previously described [16]. Data were binned into 10-min intervals for further processing.

Waveforms, periodograms and actograms were generated using the software El Temps (Dr Antoni Díez-Noguera, University of Barcelona, Barcelona, Spain). For each animal, data were smoothed with a 20min moving average and the significant (*p* ≤ 0.05) rhythms determined using the Sokolove-Bushell periodogram [17] for periods ranging between 10 and 28 h, which detects both semidiurnal and diurnal oscillations. One hour-binned mean waveforms were calculated for the group of animals in each phase of the experiment.

### General statistical methods

Data did not meet either the normality or homoscedasticity requirements for t-test or ANOVA models. For two-group comparisons, we used the Mann-Whitney test and for 3-group comparisons the Kruskal-Wallis test, followed by Mann-Whitney post hoc tests with Bonferroni's correction when appropriate. We used the Kolmogorov-Smirnov test to compare waveforms with an adjusted significance threshold of *p* = 0.05 for multiple comparisons (Bonferroni's correction) when comparing 3 curves. We conducted all the analyses either including all crabs or only rhythmic crabs (those whose periodogram showed a statistically significant ~24h periodicity) and the statistical results were similar; we only report the analysis on rhythmic crabs. All statistical analysis was done with R (version 3.2.3).

## Results

**Fig 1:**
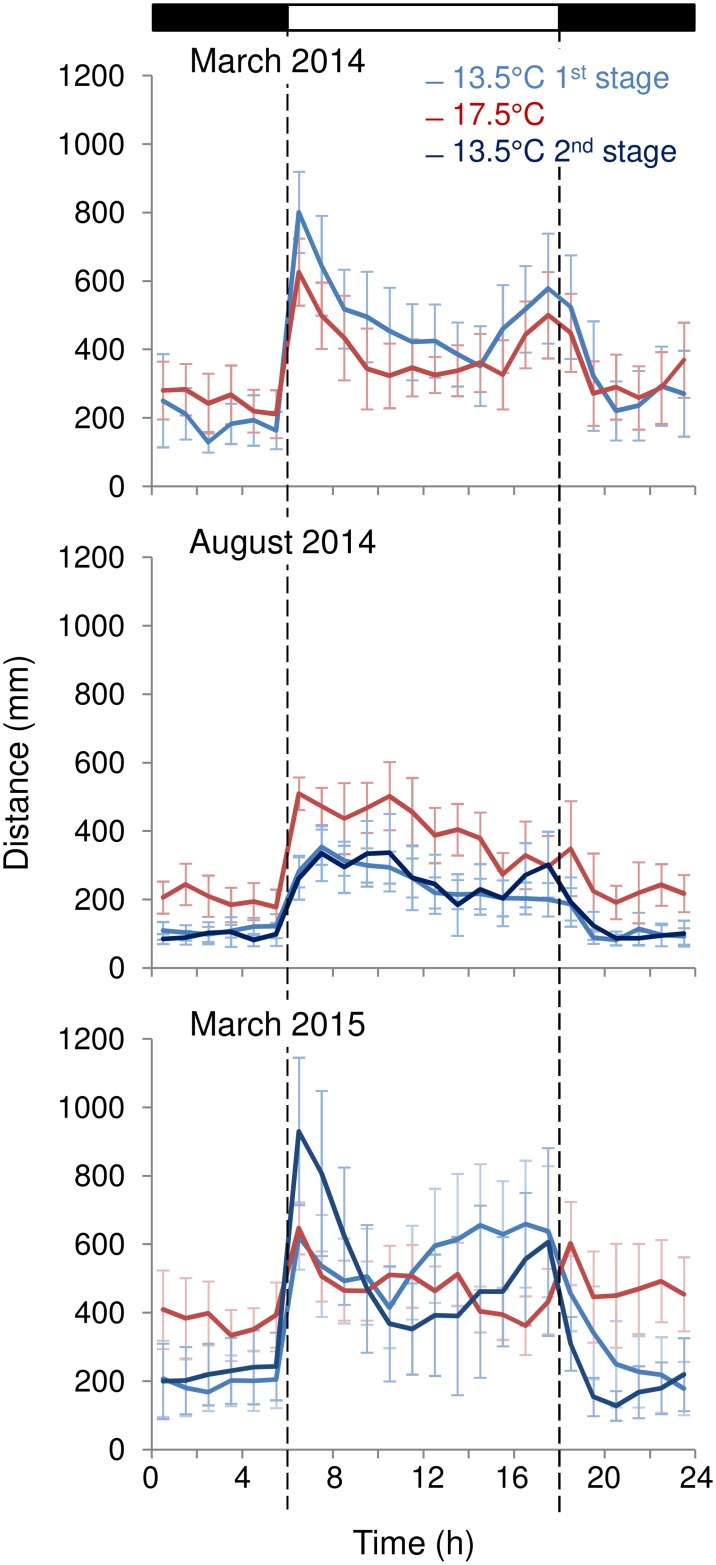
Mean waveforms for rhythmic crabs. Mean waveforms for the locomotor activity of rhythmic crabs for March 2014, August 2014, and March 2015. Mean ± SEM represent the consensus waveform that results from averaging the individual waveforms from every rhythmic crab at 13.5°C and 17.5°C. March 2014: n = 13 and 19 rhythmic crabs at 13.5°C and 17.5°C respectively. August 2014: n = 15, 15 and 10 rhythmic crabs under the three temperature conditions. March 2015: n = 17, 17 and 16 rhythmic crabs. Top bar indicates the light (white) and dark (black) phases. Dashed vertical lines mark the light on and off times.

In March 2014, the mean total distance crabs covered per day were not different between the conditions: 9050 ± 724 mm under the cold temperature (13.5°C) and 8294 ± 689 mm under the warm temperature (17.5°C) (Mann-Whitney test, *p* > 0.05, n = 13 and n = 19 rhythmic crabs, respectively). Mean waveforms were not different between conditions (Fig 1; Kolmogorov-Smirnov test, *p* > 0.05). The ratios between the distance covered under light over the distance covered under dark were 3.23 ± 0.68 at 13.5°C and 2.03 ± 0.35 at 17.5°C, showing that crabs had a significant higher amplitude rhythm under cold than warm temperature (Mann-Whitney test, *p* = 0.0302).

In August 2014, out of 22 crabs studied, there were respectively 15, 15 and 10 rhythmic individuals under the three temperature conditions (13.5°C, 17.5°C, 13.5°C; Fig 1). After the warm condition, we brought crabs back to the cold condition to determine whether the changes in activity level described represented a response to the temperature or a response to time in captivity. The total daily activity was different under the two temperatures: 4384 ± 273 mm (13.5°C), 7575 ± 573 mm (17.5°C) and 4506 ± 294 mm (13.5°C) respectively (Kruskal-Wallis test, *p* < 0.001). Post hoc comparisons indicated that the 2 cold conditions were not different (Mann-Whitney test, *p* > 0.05) while the warm condition was different from the 2 cold conditions (Mann-Whitney tests, *p* < 0.001 and *p* < 0.01 respectively). Similarly, the mean waveforms were not different at 13.5°C (Fig 1; Kolmogorov-Smirnov test, *p* > 0.05) while the curve at 17.5°C was different from the 2 curves at 13.5°C (Kolmogorov-Smirnov test, *p* = 0.039 for both). However, the ratios between the distance covered during the light phase over the distance covered during the dark phase were not different under the 3 conditions: 2.60 ± 0.28 (13.5°C), 2.22 ± 0.28 (17.5°C) and 2.82 ± 0.37 (13.5°C; Kruskal-Wallis test, *p* > 0.05), showing no difference in the amplitude of the activity rhythm.

In March 2015, out of 28 crabs studied, there were respectively 17, 17 and 16 rhythmic individuals under the three conditions (13.5°C, 17.5°C, 13.5°C; Fig 1). Similarly, to the experiment in March 2014, the mean distances covered per day in March 2015 were not different between the two temperatures (9714 ± 856 mm, 10855 ± 763 mm and 8916 ± 1377 mm respectively, Kruskal-Wallis test, *p* > 0.05). The mean waveforms for the 2 cold conditions were not different, and there was also no difference between the first cold condition and the warm one (Kolmogorov-Smirnov test, *p* > 0.05), but the second cold condition was different from the warm condition (Kolmogorov-Smirnov test, *p* = 0.015). The ratios between the distance covered during the light phase over the distance covered during the dark phase were 3.63 ± 0.55 (13.5°C), 1.58 ± 0.18 (17.5°C) and 3.44 ± 0.76 (13.5°C; Kruskal-Wallis test, *p* < 0.001). The ratios between the 2 cold conditions were not different (Mann-Whitney test, p > 0.05) but the ratio under the warm condition was different from both cold conditions (Mann-Whitney test, *p* < 0.001 for both), showing again that in March crabs had a higher amplitude rhythm under cold than warm temperature.

**Fig 2:**
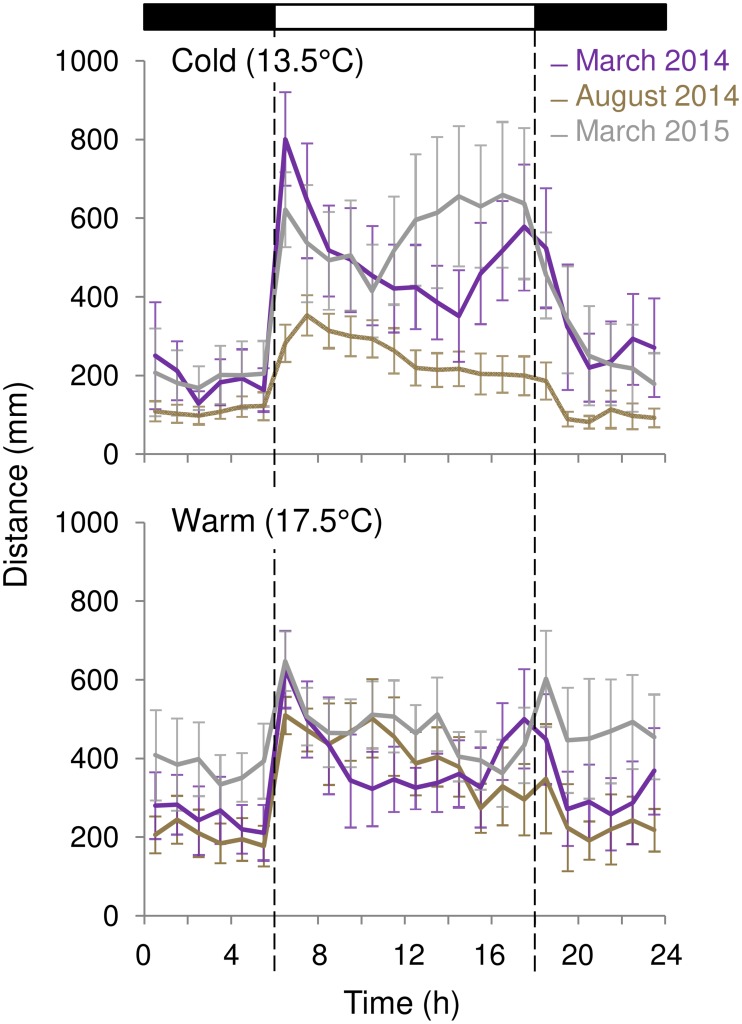
Mean waveforms throughout the experiments. Top panel: comparison of the first consensus waveforms drawn for rhythmic crabs at 13.5°C. Lower panel: comparison of the consensus waveforms drawn for rhythmic crabs at 17.5°C. Data are replotted from Fig 1. Each point represents mean ± SEM.

We compared the levels of activity between the three experiments under either the cold or warm conditions (Fig 2). For the first cold condition, the mean waveforms were not different between March 2014 and March 2015 (Kolmogorov-Smirnov test, *p* > 0.05) while the mean waveform in August was different from both March experiments (Kolmogorov-Smirnov test, *p* <0.01 for both). The level of activity at 13.5°C differed (Kruskal-Wallis test, *p* < 0.001): there was no difference between the 2 March experiments (*p* > 0.05) but the activity was higher in for both March experiments than in August (Mann-Whitney test, *p* < 0.001 for both). The comparison of the warm condition between the 3 experiments did not show a clear annual pattern: March 2014 and August were not different (Kolmogorov-Smirnov test, *p* > 0.05) while March 2015 was different from both March 2014 and August (Kolmogorov-Smirnov test, *p* < 0.001 for both).

**Fig 3:**
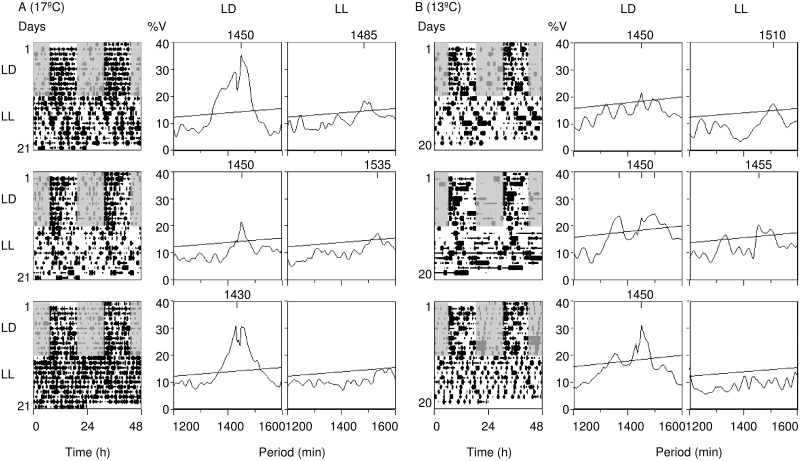
Individual actograms at 17°C and 13°C. Individual double-plotted actograms at 17°C (A) and 13°C (B) for 3 representative crabs for each experiment, kept first under a 12:12 LD cycle (10 and 8 days respectively) and then transferred to LL conditions (12 days for both). To the right of each actogram, periodograms display the period of the oscillation that significantly (*p* ≤ 0.05) explains most of the variance in the analysis, indicated in minutes. For each actogram, there are two periodograms corresponding to the activity under LD and LL, respectively. Crabs were collected in summer and tested in free-running either at 13 or 17°C. Crabs depicted under 13°C are not the same individuals depicted under 17°C.

To determine whether the rhythmic activity observed in crabs was driven by a circadian clock at each temperature, we kept crabs first under a 12:12 LD cycle and then transferred them into LL free-running conditions. Fig 3 shows 3 individual examples under the warm (left panel) and the cold (right panel) conditions. Out of 17 crabs recorded under warm conditions, 12 and 4 animals were rhythmic under LD and free-running conditions respectively. Under cold conditions, all 11 crabs were rhythmic under the LD cycle and 7 under free-running conditions. For both conditions, while some rhythmic crabs showed an apparently shorter period for the first days in free-running, all of them had a period > 24h after 3 to 4 days. The activity patterns of rhythmic crabs in LL exhibited evidence of entrainment based on phase similarity with the previous LD cycle.

## Discussion

The present study shows that shifts in environmental temperature have different effects on the daily locomotor activity rhythm of *U*. *pugilator* males depending on the time of the year. Whereas in crabs collected during the summer a decrease in temperature led to an overall decrease in locomotor activity—an expected response in an ectotherm—it led to a change in amplitude of the activity rhythm in crabs collected during the winter to early spring (i.e. early March). In other words, lower temperatures during the cold season led to respectively higher and lower levels of locomotor activity during the light and dark phases of the LD cycle. This seasonal difference in the response to temperature appears to emerge mainly from how crabs respond to cold temperatures (Fig 2). In summer-sampled crabs, cold temperature is associated with overall low levels of activity while in winter-sampled crabs the same temperature is associated with a light phase-specific increase in activity and a dark phase-specific decrease in activity.

Most studies assessing the effect of environmental temperature on biological rhythms of marine organisms have focused on the ability of temperature cycles to entrain behavioral rhythms [18–22]. Our results obtained under constant temperatures during the cold season are in line with the recently proposed circadian thermoenergetics hypothesis, which postulates that colder ambient temperatures, a decrease in food resources, or other stimuli that lead to an unfavorable energy balance shift activity to the warmer phase of the day [13, 15, 23–24]. This shift in ‘temporal niche’, the 24h temporal distribution of physiological and behavioral processes in a species [23, 25], results in a more favorable energy balance as it leads to rest during the colder part of the day when it is metabolically expensive to sustain high levels of activity. Although the hypothesis has been proposed for homeotherms it is conceivable that similar thermoenergetics trade-offs exist in ectotherms. During the winter nights, the crab’s burrow is likely warmer than the surface [12] and colder temperatures would favor more diurnal activity and low-activity retrieval to the burrow during the night. Importantly, experimental evidence shows that mice use the LD cycle as a reliable reference for the time of day and will undergo this temporal niche switch even when the low temperature is artificially held constant throughout the day [15]. In contrast to the thermoenergetic demands of the winter, the more favorable energetic balance of the summer for *U*. *pugilator* would not lead to this nocturnality/diurnality switch in response to cold temperature. Although the changes in the daily locomotor activity pattern in response to shifts in the constant environmental temperature in the laboratory support our interpretation in terms of thermoenergetic trade-offs, 24h temperature oscillations in intertidal habitats are much more complex and change seasonally. Future studies should test predictions of the circadian thermoenergetic hypothesis both in laboratory and field conditions.

Importantly, temporal niche switches seem to be much more common in nature than predicted by laboratory studies (for reviews see [23, 25–27]) and have been also reported in marine invertebrates including oysters [28] and lobsters [29]. The environmental stimuli that trigger these switches differ widely but it is likely that environmental temperature represents a key stimulus not only in homeotherms but also in ectotherms living in variable thermal environments like intertidal habitats. Studies that addressed the temperature tolerance and preference in *U*. *pugilator* males in the laboratory have shown that animals prefer the higher range of their geographic temperature spectrum and show better locomotor activity performance near these preferred temperatures [30,31]. Furthermore, *U*. *pugilator* from Florida show inactivity at extreme cold temperatures (5°C, [32]). These studies are important in establishing responses to acute changes in ambient temperatures. Our results suggest the trade-offs associated with these acute responses to environmental temperature differ between winter and summer. In other words, shifting daily activity to the warmer phase of the day, which may be used to facilitate locomotor activity in ectotherms [23], may be a response that is specifically favored during the winter. It remains to be determined whether the winter/summer variation in *U*. *pugilator*’s response to temperature changes represents a response to seasonal environmental cues such as photoperiod length or the output of a circannual clock [33].

*U*. *pugilator* are low-tide active crabs that typically retrieve to their burrows during high tides [10]. Barnwell (1966) reported that *U*. *pugilator* collected from the Woods Hole region showed prominent circatidal rhythmicity [8], and this has been confirmed in other *Uca* species [3, 4]. We did not observe any obvious circatidal components in our recordings likely because tests were not done immediately after animal field collection, and crabs were held under LD entrainment only. The detection of circatidal rhythmicity in intertidal species is highly variable both within and between intertidal species. Whereas some species show robust free-running rhythms for several months under constant laboratory conditions [34–35] others present very labile rhythms that are only present in isolated individuals [4, 36–37]. Previous studies in *U*. *pugilator* also reported more robust nocturnal activity than we described here [8, 38]. At least two reasons could account for this difference. First, fiddler crabs from the same species sampled from coastal habitats with different tidal regimes can have strikingly different patterns of rhythmic locomotor activity [35]. Second, previous experiments were done under higher temperatures (~25°C), which could have favored more nocturnal activity.

The locomotor activity rhythm measured under both cold and warm conditions presents at least two of the properties of bona fide circadian rhythms: i) the rhythmic activity persists under constant conditions with a period close to 24 h, ii) the rhythm is entrained by the LD cycle. Importantly, in crabs with robust rhythmicity under LL conditions the phase of the activity rhythm matched the phase before transition into LL, confirming that the rhythm is truly entrained by the LD cycle [39, 40]. In this sense, the circa-24h locomotor activity rhythms we measured in *U*. *pugilator* likely represent true circadian rhythms (with periods close to 24 h and entrained by 24h environmental cycles) and not circalunidian rhythms (with periods close to 24.8 h and entrained by lunar day-associated environmental cycles [4]). Importantly, due to the phase instability and the lack of clear phase markers in the rhythms of locomotor activity, our phase assessment is based on visual inspection of actograms rather than in a mathematical measurement of phase under LD and LL conditions. Not all crabs remained rhythmic under constant illumination. This is consistent with previous reports indicating that rhythms in marine organisms can be labile in the laboratory, particularly under free-running conditions [4, 36, 41]. All rhythmic crabs showed a period exceeding 24 h, consistent with previous observations in *U*. *pugilator* under constant illumination [8].

In conclusion, we show that the daily behavioral responses to changes in environmental temperature in *U*. *pugilator* differ depending on the time of the year, and that—as in other species—nocturnality and diurnality are not fixed, absolute states but rather the result of flexible biological timing systems that have evolved in response to complex temporal environments. This seasonality could modulate the sensitivity and tolerance to temperature changes as well as the temporal distribution of behaviors of these sand dwellers. Ectotherms are believed to be more sensitive to climate change [42] and the intertidal habitat appears to be particularly sensitive to global environmental changes [43]; studies on the physiological and behavioral responses of intertidal species induced by temperature changes will be critical to understand and model the resilience of these ecosystems in a global warming scenario.

## Supporting information

S1 Dataset Locomotor activity data. (7Z)
